# Combining Temporal and Spectral Information with Spatial Mapping to Identify Differences between Phonological and Semantic Networks: A Magnetoencephalographic Approach

**DOI:** 10.3389/fpsyg.2012.00273

**Published:** 2012-08-09

**Authors:** Fiona McNab, Arjan Hillebrand, Stephen J. Swithenby, Gina Rippon

**Affiliations:** ^1^Wellcome Trust Centre for Neuroimaging, Institute of Neurology, University College LondonLondon, UK; ^2^Department of Clinical Neurophysiology and Magnetoencephalography Center, VU University Medical CenterAmsterdam, Netherlands; ^3^Faculty of Science, Departments of Life, Health and Chemical Sciences, The Open UniversityMilton Keynes, UK; ^4^Aston Brain Centre, School of Life and Health Sciences, Aston UniversityBirmingham, West Midlands, UK

**Keywords:** magnetoencephalography, synthetic aperture magnetometry, beta, gamma, phonological processing, semantic processing, beamforming

## Abstract

Early, lesion-based models of language processing suggested that semantic and phonological processes are associated with distinct temporal and parietal regions respectively, with frontal areas more indirectly involved. Contemporary spatial brain mapping techniques have not supported such clear-cut segregation, with strong evidence of activation in left temporal areas by both processes and disputed evidence of involvement of frontal areas in both processes. We suggest that combining spatial information with temporal and spectral data may allow a closer scrutiny of the differential involvement of closely overlapping cortical areas in language processing. Using beamforming techniques to analyze magnetoencephalography data, we localized the neuronal substrates underlying primed responses to nouns requiring either phonological or semantic processing, and examined the associated measures of time and frequency in those areas where activation was common to both tasks. Power changes in the beta (14–30 Hz) and gamma (30–50 Hz) frequency bands were analyzed in pre-selected time windows of 350–550 and 500–700 ms In left temporal regions, both tasks elicited power changes in the same time window (350–550 ms), but with different spectral characteristics, low beta (14–20 Hz) for the phonological task and high beta (20–30 Hz) for the semantic task. In frontal areas (BA10), both tasks elicited power changes in the gamma band (30–50 Hz), but in different time windows, 500–700 ms for the phonological task and 350–550 ms for the semantic task. In the left inferior parietal area (BA40), both tasks elicited changes in the 20–30 Hz beta frequency band but in different time windows, 350–550 ms for the phonological task and 500–700 ms for the semantic task. Our findings suggest that, where spatial measures may indicate overlapping areas of involvement, additional beamforming techniques can demonstrate differential activation in time and frequency domains.

## Introduction

Mapping the neural correlates of different language functions has a long and detailed history in cognitive neuroscience, most recently addressed by the application of brain imaging techniques (e.g., Salmelin and Kujala, 2006). One key issue is that different brain imaging modalities have mainly focused on separate questions, with PET/fMRI studies addressing the “where” issue and EEG/ magnetoencephalography (MEG) studies highlighting “when” information within a less well-defined spatial mapping framework (Pulvermüller et al., 2003). Relatively few studies have combined these approaches comprehensively (Indefrey and Levelt, 2004; Pulvermüller et al., 2006) or explored the additional insights that can be gained by considering the functional significance of variations in oscillatory neuronal activity (Bastiaansen et al., 2010).

One sphere in language research in which there has been a focus on the presence or absence of functional parcellation and segregation of specific roles within common cortical areas is that of phonological and semantic processing (see e.g., review Vigneau et al., 2006). The introductory overview below of the separate spatial, temporal, and spectral approaches in this field highlights some of the difficulties in resolving the structures and related functions involved in accomplishing these distinct cognitive tasks. The body of the paper will then demonstrate how MEG can be utilized to resolve some of these issues.

### Spatial mapping of phonological and semantic processing

Early research based on lesion studies suggested a relatively straightforward segregation of neural correlates of phonological and semantic processing, with the superior/middle temporal cortex identified as the location of so-called “semantic stores” (Patterson et al., 2007) and the inferior parietal lobule as the site of the “phonological store” (Paulesu et al., 1993). Frontal areas, most specifically the inferior frontal gyrus (IFG) or Broca’s region, were associated with language production or the motor components of speech. However, increasingly sophisticated brain mapping techniques have revealed the flaws in this “functional parcellation” approach. A meta-analysis by Vigneau et al. (2006) revealed a marked overlap of “semantic” and “phonological” clusters in the middle and inferior temporal gyri, although a possible differentiation of a dorsal “sound-based processing” component based around the auditory cortex was proposed. A recent review of neuroimaging studies of the “phonological store” (Buchsbaum and D’Esposito, 2008) has thrown doubt on its location in parietal areas, identifying additional activation of temporal areas, such as the involvement of posterior temporal cortex (including Wernicke’s area) in phonological code retrieval (for example, de Zubicaray et al., 2002; Heim and Friederici, 2003). In parallel, a meta-analysis of 120 fMRI studies by Binder et al. (2009) has identified consistent reports of parietal involvement in semantic processing, although the location, in the angular gyrus or the temporo-parietal junction, does not place it firmly in the parietal area. Syntactic, semantic, and phonological processes have all been identified within the IFG (see reviews, Bookheimer, 2002; Vigneau et al., 2006), although considerable debate exists as to the exact nature of the process involved, with strong claims that the apparent involvement is due to task demands rather than linguistic processing *per se* (e.g., Binder et al., 2009). Hagoort (2005) has proposed a “unification” role for the left IFG, bringing together the different types of language information; phonological (including prosody and syllabic structure), syntactic, and semantic structure. However, a contradictory proposal by Grodzinsky and Santi (2008) and a rejoinder by Willems and Hagoort (2009) indicate that “the battle for Broca’s region” has not yet been resolved.

Although some of the issues associated with this lack of consensus may be associated with insufficient attention to task confounds and task demands (Thompson-Schill, 2003; Binder et al., 2009), it is probable that the precision offered by the employed imaging techniques is insufficient to be able to distinguish between different roles for closely overlapping functional units, nor the sequence of their involvement. Although a high degree of spatial resolution is offered by fMRI techniques, limitations should be acknowledged (Logothetis, 2008; Buxton, 2012). A recent paper (Fedorenko et al., 2011) has reported an “individual differences” approach to fMRI data that can identify a high degree of functional specificity in key cortical regions when comparing linguistic with non-linguistic cognitive processes. However, this type of approach has not yet been used to demonstrate such segregation between the networks underpinning different linguistic processes. Another way of attempting such segregation could be to harness the high-level of temporal resolution and additional frequency information offered by techniques such as MEG.

### Temporal tracking of phonological and semantic processing

Given the brief time course involved in language processing, disentangling the alleged overlapping or non-overlapping functions of key areas in the language networks cannot rely on the relatively poor temporal resolution of techniques based on the hemodynamic response, whereas techniques such as EEG and MEG, with greater time resolution, can assist in this process (Pulvermüller et al., 2003).

ERP/ERF paradigms (event-related potentials/fields) have been employed to generate a word processing “time-line.” The common protocol in such studies is the use of a “violation” technique, where an expectation as to the phonological or semantic characteristics of a word is established within a sentence, with this expectation then violated by the target word (e.g., “Robin Hood stole from the rich and gave to the porcupine”). Phonological Mismatch Negativity’ (PMN) paradigms place phonological processing at about 200–350 ms post-stimulus presentation (e.g., Connolly and Phillips, 1994; Kujala et al., 2004). Semantic processing, similarly assessed by the so-called “semantic incongruity” paradigms’ (Kutas and Hillyard, 1980) is associated with a response at about 400 ms after word onset. This is known as the N400 response. The amplitude of this response can be modulated by the degree of expectancy established by the context, the N400 effect. Semantic priming is another technique used to illustrate semantic expectancy effects. Using word pairs, it has been shown that the response to the target word is faster and the N400 smaller if the target word is semantically related to the prime word (Kutas and Feidermeier, 2011). Reviews of such studies have allowed for confident tracking of the evolution of word and sentence processing over time (Salmelin and Kujala, 2006; Salmelin, 2007).

It could be assumed that examining the combined topographical and timing information contained in the ERP language components would help to resolve the discrepancies reported in studies that are solely based on spatial information. Accurate source localization of EEG responses themselves can be problematic due to signal distortion but simultaneous fMRI-EEG/ERP techniques have been developed to allow accurate identification of the cortical areas associated with EEG/ERP responses. However, this spatio-temporal mapping approach, although broadly confirming the above time-line, has not proven decisive in identifying distinct neural correlates for phonological and semantic processing. The N400 has been most reliably located in the predicted temporal areas using combined fMRI and ERP (Matsumoto et al., 2005) or MEG (Kujala et al., 2004). The left IFG has also been implicated in some studies (Ruff et al., 2008) but not in others (Van Petten and Luka, 2006). Maess et al. (2006), using MEG, identified six spatially separate N400 sources, including left temporal regions with left IFG activated by semantic violations. A review by Lau et al. (2008) locates the N400 source in the middle temporal gyrus, suggesting that IFG involvement is associated with task demands such as information retrieval and response mediation rather than semantic processing *per se*. The PMN has not been located in predicted parietal areas but instead in left temporal regions overlapping those reported for the N400 (Indefrey and Levelt, 2004; Kujala et al., 2004). It has, in fact, been suggested that the PMN and N400 are not genuinely separable (Van den Brink et al., 2001; Van den Brink and Hagoort, 2004) and are part of a continuous process starting at about 200 ms and continuing for some 300–350 ms (Van den Brink and Hagoort, 2004; Bonte et al., 2006).

In addition, it should be noted that the use of average evoked activity, which reflects activity that is phase-locked to the stimulus across trials, may disguise the inherent variability of higher level cognitive processes. Induced components which are time-locked but not necessarily phase-locked would not be revealed by averaging in the time domain (Tallon-Baudry and Bertrand, 1999).

However, trial by trial analysis in the frequency domain can reveal event-related changes in power; these can be localized subsequently to their sources using, for example, beamformer techniques (Pammer et al., 2004; Hillebrand et al., 2005). This therefore adds the possibility of examining oscillatory responses as dependent variables in attempts to differentiate the contributions of different cortical areas to language processing.

### Oscillatory activity associated with phonological and semantic processing

Relatively few studies have harnessed the spectral information available in EEG/MEG recordings in order to further characterize the neural underpinnings of phonological/semantic processing. Emerging evidence of the different functional roles of distinct frequencies together with the insights they may offer into the transient coupling and uncoupling of relevant neural networks (Varela et al., 2001) suggest that this approach may have much to contribute to the debate.

A recent paper by Kujala et al. (2011) looking at phonological and semantic priming, suggested that aspects of word processing related to phonological content were associated with interactions at higher frequencies (specifically 66 Hz, i.e., in the gamma frequency range) whereas analysis of word meaning utilized interactions in lower frequencies (8 Hz or within the alpha range). Conversely, Braeutigam et al. (2001) reported variations in phase-locked gamma associated with semantic incongruity and Hald et al. (2006) described increases in induced frontal gamma activity also associated with semantic violations. Shahin et al. (2009) suggest that theta, upper beta as well as gamma activity are all associated with template matching activities supporting semantic evaluation. In a review of the field, Bastiaansen et al. (2010) suggested that beta and gamma oscillations index the “unification” aspect of language comprehension, with beta oscillations associated with syntactic unification and/or resolution of syntactic complexity and gamma oscillations associated with higher sentence-level semantic processing and “reconciliation.” This latter suggestion parallels the general “binding” role attributed to gamma activity (Tallon-Baudry and Bertrand, 1999; Fries et al., 2007), not only in sensory processing but also in various cognitive processes (Jensen et al., 2007). This is supported in a study by Hannemann et al. (2007) using a degraded speech word identification task, where only correctly identified items were associated with significant enhancement of induced 40 Hz gamma band activity (GBA). The enhancement was not seen with evoked measures.

A more recent study by Obleser and Kotz (2011) that used EEG to measure N400 and gamma activity in a degraded speech sentence task demonstrated that, while the N400 showed the expected variation with the predictability of the final word, i.e., a larger response to a less predictable word, induced GBA was greater the more predictable the final word. The authors suggest that the N400 reflects the semantic processing of the stimulus, whereas the gamma activity indexes the “satisfactory” resolution of the sentence as a whole. This is supported by the later time window for the gamma response, at ∼600 ms Hannemann et al.’s (2007) single word protocol was associated with GBA at left temporal electrode sites, whereas the GBA in the Obleser et al. study had a left fronto-temporal-central distribution.

Changes in other frequency bands have been associated with more indirect aspects of word processing tasks. Activity in the theta band has been associated with engagement of verbal working memory (Bastiaansen et al., 2010) or with retrieval processes (Hald et al., 2006). Klimesch et al. (1997), using a semantic congruency paradigm, suggest that activity in the alpha frequency band may not separately index different phases of linguistic processing but may be related to more generic task demands, probably based around judgment and decision-making. Taken separately, then, these different approaches to mapping brain activity have had limited success in segregating the neural correlates of the phonological and semantic components of language processing. We propose to use a combination of the approaches by analyzing location, timing, and spectral signatures of key events. Taken together, these may not only identify the structures underlying both processes, but also allow for discrimination between different functional roles played by structures that are apparently common to both language components. We are not suggesting that the spatial resolution alone of our approach can match that shown by fMRI but that harnessing the superior temporal resolution and additional spectral information that can be offered by beamforming approaches (Singh et al., 2002; Hirata et al., 2004; Hall et al., 2005; Hillebrand and Barnes, 2011) could offer the kind of “fine-grained” characterization of different components of cognitive processes that is not available to techniques based on slower hemodynamic activity (Logothetis, 2008; Buxton, 2012). It is also the case that beamforming methods can offer powerful source localization solutions, showing strong correlations with fMRI data itself, with anatomical measures with animal data and with invasive recordings (Singh et al., 2002; Hirata et al., 2004; Hall et al., 2005; Hillebrand and Barnes, 2011).

In a previous paper (McNab et al., 2007), we reported on the application of such an approach in a study of task priming. We used a beamformer technique (Synthetic Aperture Magnetometry, SAM) to measure the spatial, temporal, and spectral characteristics in response to single words that primed either phonological or semantic processing of target words. Our main focus there was on the characterization of responses to the task-primes and within-task matching to their targets. Here we focus on cross-task comparisons of responses to the target stimuli, to examine the extent to which a combination of spatial, temporal, and spectral measures could separate out the neural correlates of phonological and semantic processing. As outlined above, it is clear that although phonological and semantic processing of language are clearly differentiable aspects of linguistic analysis, which can be separately modulated, for example by cortical damage, individual brain imaging techniques have struggled to produce distinct cortical maps for the two processes. We feel that the data examined here offer the possibility to (a) demonstrate that behaviorally distinct language processes may elicit activity in spatially identical or closely overlapping cortical areas but (b) examination of the temporal and/or spectral characteristics of that activity can provide means of distinguishing the contribution of these areas to the separate processes. Unusually, therefore, our focus will be on those areas where significant differences between task and baseline are found for both tasks, rather than on areas which are only activated by one task or the other.

Frequency analysis will be restricted to beta and gamma bands as (a) these have been specifically associated with phonological and semantic processing (Bastiaansen et al., 2010; Kujala et al., 2011) and (b) a number of studies have indicated a tight correlation between the BOLD signal and gamma/beta LFP (Logothetis et al., 2001; Mukamel et al., 2005; Shmuel et al., 2006). Given that our aim is to differentiate task-specific processes in regions that have been implicated in both tasks by BOLD/fMRI studies, and the relatively brief time windows associated with these types of linguistic tasks, we have focused our analyses on these higher frequency bands.

Time windows will be selected on the basis of those associated with the time-lines for comparable phonological and semantic processing (Indefrey and Levelt, 2004; Kujala et al., 2011).

We predict that both processes will elicit activity in the temporal areas, as this is a commonly reported finding with both spatial and temporal mapping techniques (Vigneau et al., 2006).

Parietal areas have more reliably been associated with phonological processing (Paulesu et al., 1993), although it has been suggested that semantic processing may automatically engage phonological processing in parallel (Nobre and McCarthy, 1994; Matsumoto et al., 2005). Similarly, Binder et al.’s (2009) review has identified the angular gyrus or temporo-parietal junction as part of a semantic network. It is possible, therefore, that co-location of activation by both processes will occur in the parietal area.

Similarly, although frontal areas are classically associated with phonological processing (Paulesu et al., 1993), engagement during semantic processing as well is a continuing matter of debate (Binder et al., 2009; Willems and Hagoort, 2009). Most studies demonstrating frontal involvement with semantic processing involve higher level sentence processing as opposed to the primed single word processing task employed here. We predict therefore that, while frontal activation may be associated with the phonological task, it will not be reliably associated with the semantic task.

## Materials and Methods

The data and analysis approach were as described in McNab et al. (2007). Eleven participants gave informed consent to take part in this study (six females). All stated themselves to be right handed native English speakers, with normal or corrected to normal vision.

### Stimuli

One hundred and twenty-eight nouns were visually presented, including 64 names of living things (of which 32 had one syllable and 32 had two syllables) and 64 names of non-living things (of which 32 had one syllable and 32 had two syllables). Words from each of these four categories were matched in terms of number of letters (three–nine letters, mean word length = 5.2, σ = 1.6) and word frequency score (Kucera and Francis, 1967) obtained from the MRC Psycholinguistic Database (1987; maximum word frequency = 114, mean word frequency = 11.5, σ = 21.2).

### Behavioral study

In order to validate the assumed distinctiveness of phonological and semantic processing within the two task conditions, a behavioral study was conducted which used an incidental learning paradigm and produced results in keeping with the “level of processing” memory effect (Craik and Lockhart, 1972). Sixty-nine percent of words that had been semantically encoded (with the living/non-living task) and 60% of words that had been phonologically encoded (with the syllable counting task) were correctly recognized in a later recognition phase in which all 128 encoded words were presented together with 128 new words. The new words were matched to the encoded words in terms of number of syllables, whether they were living or non-living, word frequency and number of letters. A one-way within-subjects ANOVA on the recognition scores revealed a significant main effect of task type [*F*(1, 96) = 9.342, *p* < 0.01].

### Procedure

**Figure 1:**
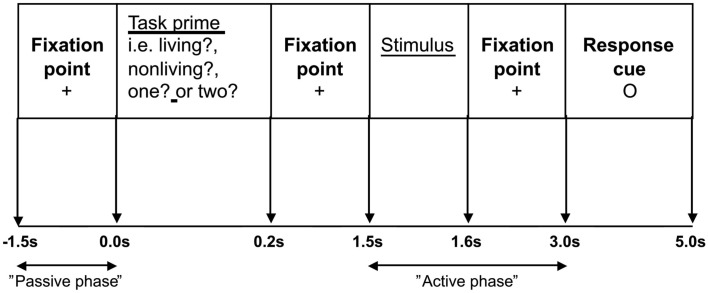
**The stimulus sequence: Participants were asked to fixate on a cross in the center of the screen for 1.5 s**. The task-prime was then displayed for 0.2 s, followed by a fixation cross for 1.3 s. The target stimulus was then presented for 0.1 s, the fixation cross was shown for 1.4 s before the response cue (a small circle in the center of the screen) was displayed for 2.0 s.

Participants were shown the nouns on a computer monitor which was viewed directly through a window in the MEG shielded room. There were two tasks. A semantic judgment task required participants to decide whether the word referred to a living or non-living entity and a phonological task involved syllable counting. Each word was presented twice; once within each of the two task conditions. Trials were presented in pseudo-random order, so that one task condition did not involve more repeated words than the other. Prior to stimulus onset, participants were informed which task to perform by the presentation of a task-prime or “prompt,” which took the form of a single word question (“living?”, “non-living?”, “one?”, and “two?”). All words were presented in black, on a white background, with a single line frame around the stimuli in order to differentiate them from the task-primes/prompts. The different types of nouns were evenly assigned to the different task-prime conditions. Primed/prompted yes/no responses were made via a button press with the left index finger. The stimulus sequence is represented in Figure 1.

Following a 1.5 s prestimulus period, a task-prime/prompt appeared for 0.2 s informing participants which task to perform when the stimulus appeared at 1.5 s. Participants were asked to wait until a response cue appeared at 3.0 s before making their response. Prior to scanning participants completed a practice session of seven trials.

### MEG recording and co-registration with MRI data

Magnetoencephalography data were collected using a 151-channel CTF Omega system (CTF Systems, Inc., Port Coquitlam, Canada). The data were collected in third order mode, at a sampling rate of 625 Hz. Following data acquisition the shape of the participant’s head was digitized using a 3D digitiser (Polhemus Isotrak). This surface was matched to that extracted from the participant’s anatomical MRI, using Align^1^, so that the MEG data obtained from each participant could be co-registered with a previously acquired anatomical MRI scan (Adjamian et al., 2004). A head-shape was subsequently extracted from the co-registered MRI, and a multi-sphere model was created on the basis of this head-shape (Huang et al., 1999). This multi-sphere head model was used to model the volume conduction for the beamformer analysis (see below).

### Data analysis

A bandpass filter (0.7–80 Hz) and a 50 Hz power line Butterworth filter were applied to the data, and D.C. offset was removed. Epochs containing eye blink artifacts were identified by visual inspection, and omitted from further analysis. The data were analyzed using SAM, which is an adaptive beamforming technique whereby each voxel in the brain is linked to the detection array using an optimal spatial filter (Robinson and Vrba, 1999; Vrba and Robinson, 2001; Hillebrand et al., 2005). In this way we were able to produce 3D spatial images of spectral power change between predefined active and passive time windows (see below). These pseudo-*T* images include a depth weighting (Vrba and Robinson, 2001; Hillebrand and Barnes, 2005). Noise regularization was not applied. SAM analysis enabled us to increase the signal to noise ratio (with the weights acting as a spatial filter; Hillebrand et al., 2005; Hoogenboom et al., 2006). Details of the sensitivity of our MEG system to signals from different brain regions can be found in Hillebrand and Barnes (2002). Although the spatial resolution of beamformer images can be very high (∼1 mm; Hillebrand and Barnes, 2005), it is generally in the order of several millimeters (Barnes et al., 2004) depending on factors such as the source strength, number of sensors, and MEG-MRI co-registration accuracy (Hillebrand and Barnes, 2003). In this study we used a grid-spacing of 5 mm for the analysis.

Both direct and indirect comparisons were performed. Direct comparisons involved treating the semantic condition as the “active” phase and the phonological condition as the “passive” phase. Indirect comparisons involved comparing either the semantic condition or phonological condition (“active phase”) with a prestimulus baseline.

For each condition separately, SAM analyses were conducted using time windows of 350–550 and 500–700 ms as the active phase and 200 ms of prestimulus time as the passive phase. These time windows were selected on the basis of those associated with the time-lines for comparable phonological and semantic processing (Indefrey and Levelt, 2004; Kujala et al., 2011). The data for these time windows in the 64 trials were combined to construct the data covariance matrix for each (active and passive) phase, which were used to construct the beamformer weights. The reconstructed source power for the active and passive phase were subsequently contrasted, resulting in, for each condition and for each participant, a 3D statistical parametric map (beamformer image). The above was repeated for each of the different frequency bands (14–20, 20–30, 30–40, and 40–50 Hz).

Using Statistical Parametric Mapping (SPM99, Friston et al., 1995), individual beamformer images were spatially normalized and averaged to produce a group image, referred to as group SAM (Singh et al., 2002). These group images were visualized using mri3dX^2^ and their statistical significance assessed using statistical non-parametric permutation testing, SnPM (Singh et al., 2003). Only results significant at the *p* < 0.05 level are reported here. The SnPM procedure employed included a multiple comparison correction which uses a probability distribution generated by the largest pseudo-*T* values in the volume, instead of using the pseudo-*T* value at each voxel (see Singh et al., 2003 for details). No correction was made for the number of time windows and frequency bands analyzed and no cluster-level analysis was performed.

In order to look at spectral profiles in detail, a so-called virtual electrode analysis was performed, where time-series of activation were reconstructed for voxels corresponding to regions of the template brain that showed a statistically significant effect at the group level (Singh et al., 2002; Barnes and Hillebrand, 2003; Hall et al., 2004). Subsequently, Morlet wavelet analysis (using a wavelet width of 7) was performed on these time-series in order to obtain time-frequency representations for each condition, which were subsequently compared using a Mann–Whitney analysis in order to identify time/frequency effects of the task at the individual participant level.

## Results

Participants completed the task with a mean accuracy of 98.0% (σ = 1.2%) for the semantic task, and 97.6% (σ = 2.4%) for the phonological task.

Only baseline-task comparisons showed significant differences. The co-ordinates of each of the peak voxels that showed significant effects (*p* < 0.05, corrected) for the comparisons between semantic and phonological task conditions and their respective prestimulus baseline are presented in Table 1.

**Table 1:** **The co-ordinates of each of the peak voxels that showed significant effects (*p* < 0.05, corrected) for the two task conditions relative to their respective prestimulus baseline, identified using Statistical Non-Parametric Mapping, for the two time windows**.

Frequency	350–550 ms		500–700 ms	
	Semantic	Phonological	Semantic	Phonological
14–20 Hz	Right superior temporal gyrus, 51, −12, −3	Right cerebellum, 30, −87, −48	–	Left inferior frontal gyrus near BA45, −57, 24, 12
		Left occipital lobe next to BA37 middle temporal gyrus, −60, −72, 3		Right fusiform gyrus BA37, 51, −48, −21
				Right cerebellum, 48, −54, −38
20–30 Hz	Left middle temporal gyrus BA39, −36, −75, 15; −54, −78, 24	Left inferior parietal lobule BA40, −36, −48, 39, −45, −60, 54	Left inferior parietal lobule BA40, −51, −66, 48	–
	Left parietal precuneus, −3, −63, 33; −12, −87, 54			
30–40 Hz	–	Left middle frontal gyrus near BA6, −27, 0, 63	–	Right parietal postcentral gyrus near BA2, 60, −30, 51
		Right precentral gyrus frontal BA44, 57, 9, 9		Left middle frontal gyrus near BA10, −39, 39, 30
40–50 Hz	Left superior frontal gyrus BA10, −15, 69, 33	–	–	–

**Figure 2:**
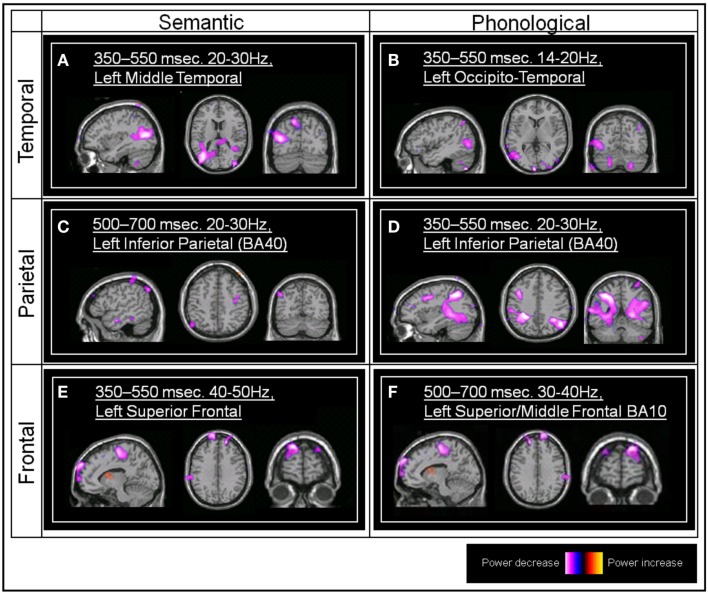
**Group SAM images**. **(A)** The significant left middle temporal effect associated with the semantic task (Talairach coordinate, TC: −36, −75, 15, and −54, −78, 24, peak pseudo-*T* value = −4.92) **(B)** The left occipito-temporal effect associated with the phonological task (TC: −60, −72, 3, pseudo-*T* value = −3.06). **(C)** The left inferior parietal effect associated with the semantic task (BA40; TC: −51, −66, 48, pseudo-*T* value = −3.71). **(D)** The left inferior parietal effect associated with the phonological task (TC: −36, −48, 39, pseudo-*T* value = −4.36). **(E)** The left superior frontal effect associated with the semantic task (TC: −15, 69, 33, pseudo-*T* value = −3.94). **(F)** The left superior/middle frontal (BA10) effect associated with the phonological task (TC: −39, 39, 30, pseudo-*T* value = −4.31).

We focus here on the frontal, parietal, and temporal regions of interest, hence significant effects within voxels lying within sensorimotor regions such as BA2 and BA6 were excluded from further analysis, as were regions lying outside those of interest to the study (for example the cerebellum). Additionally, we will only report here on those areas where significant differences between task and baseline are found for both tasks (see Figure 2).

Both the semantic and phonological tasks were associated with a statistically significant group power decrease within the left middle temporal gyrus, in the region of BA37/BA39 (all between 350 and 550 ms post-stimulus onset, and within beta frequency bands).

Both the phonological and the semantic task conditions were also associated with a statistically significant power decrease within the left inferior parietal lobule, BA40, although this occurred earlier within the phonological task (350–550 ms) compared to the semantic task (500–700 ms). Both of these effects occurred within the 20–30 Hz beta frequency band.

Both semantic and phonological task comparisons revealed a significant power decrease within left superior/middle frontal regions (BA10). This occurred earlier in the semantic task (350–550 ms) compared to the phonological task (500–700 ms). These effects were confined to the gamma frequency range, 30–40 Hz in the case of the phonological task, and 40–50 Hz in the case of the semantic task).

A statistically significant left inferior frontal (BA46) power decrease emerged from the phonological versus prestimulus comparison (14–20 Hz, 500–700 ms). Individual SAM images also showed left inferior frontal peaks within the semantic versus prestimulus comparison although this effect did not reach significance at the group level, possibly due to individual variability in both the location of these effects and the frequency band in which they were observed.

**Figure 3:**
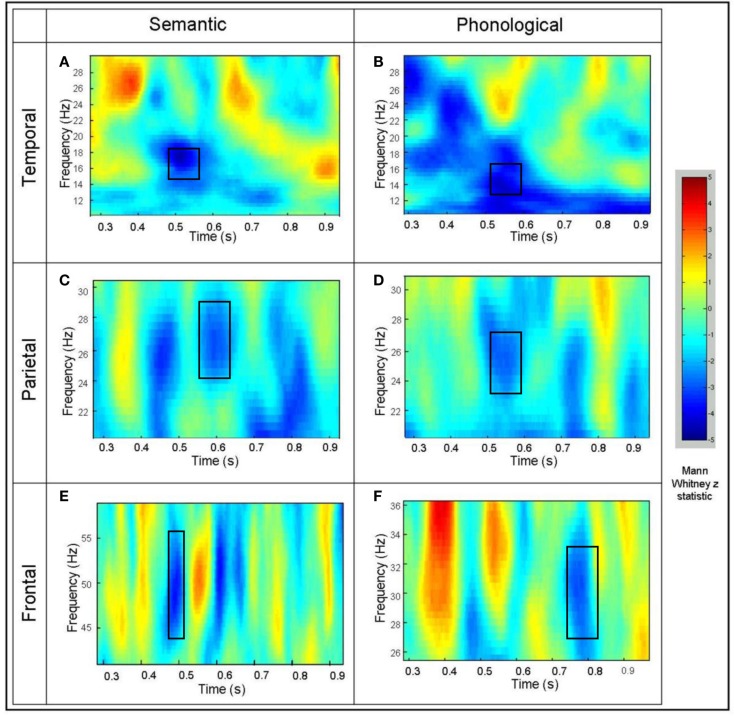
**Example time-frequency Mann–Whitney representations for individual participants for virtual electrodes placed at the peak of the power decrease seen at the group level**. **(A)** The left middle temporal effect for the semantic task and **(B)** the phonological task. **(C)** The inferior parietal effect for the semantic task and **(D)** the phonological task. **(E)** The left superior frontal effect for the semantic task and **(F)** the phonological task. The color represents the Mann–Whitney *Z* statistic for comparisons between the task and the prestimulus baseline.

In order to illustrate the time course of the beta and gamma activity in the identified areas of interest, exemplar time-frequency representations for activity from a “virtual sensor” were plotted for an individual participant (Singh et al., 2002; Barnes and Hillebrand, 2003; Hall et al., 2004). Mann–Whitney time-frequency representations are produced from the virtual electrode output, and peak values of the Mann–Whitney *Z* statistic identified in order to investigate time/frequency effects at the individual participant level (see Figure 3). The superimposed squares represent those areas where *Z* statistic peaks for the individual coincide with those that were identified in the group SAM analyses as significantly different from baseline in both tasks. It should be noted that individual TFRs may suggest areas of significant cortical activation that do not occur across the group (and thus disappear in group analyses). However, any areas of activation that are sufficiently consistent across all individuals will result in statistically significant changes as illustrated in Figure 2.

## Discussion

The focus of our analysis was on those areas where power changes were shown for both the phonological and for the semantic task. We wished to demonstrate that, although the activity was apparently spatially coincident, examination of the temporal and/or the spectral characteristics of this activity could allow a more fine-grained analysis of the precise role of these areas in phonological and semantic processing. We predicted spatial overlap for both processes in the temporal and parietal regions and examined beta and gamma activity in pre-selected time windows of 350–550 and 500–700 ms, these frequency bands and time windows having been most reliably associated in previous research with segregation of phonological and semantic processing (Indefrey and Levelt, 2004; Bastiaansen et al., 2010; Kujala et al., 2011).

Direct comparisons were not significant. This could be attributed to the focus on those areas which were associated with activation in both tasks, i.e., involving responses in closely overlapping, if not, identical members of the separate networks. It could also be a function of the acknowledged lower spatial resolution of our technique. Thus it could be anticipated that, even with techniques which, in principle, provide additional temporal and spectral information, direct comparisons illustrate the same difficulty caused by the apparent spatial coincidence of the sources of the power change, particularly where the differences are small. Since a temporal window is initially used with beamforming in order to compute the beamformer weights and to reconstruct source power for such a time window, any differences in timing between conditions is ignored, resulting in non-significant differences when performing a direct comparison between conditions (when there are no overall power differences). Only subsequent time-frequency analysis enables the identification of such subtle differences between conditions.

All the effects observed within this study arose from a power decrease associated with the task condition relative to the prestimulus interval. This is commonly reported in priming studies where a “task-set” has been established via a preceding stimulus (Matsumoto et al., 2005; Kujala et al., 2011). A power decrease in low frequencies, such as the alpha and beta bands, has been described as representing the correlate of an activated cortical area (Pfurtscheller, 2001; Klimesch et al., 2007) and there have been observations of power decreases within a range of cognitive paradigms (Dujardin et al., 1995; Singh et al., 2002). Singh et al. (2002) reported a coincidence between regions of alpha/beta power decrease and hemodynamic responses using BOLD fMRI, supporting the notion that a low-frequency power decrease represents increased neural activation. Although few other studies have used SAM analysis to investigate language processing, language processes have been associated with beta and gamma power decreases (Hirata et al., 2002; Ihara et al., 2003). Furthermore, Hirata et al. (2004) showed that language dominance estimated from power decreases observed with SAM was consistent with that determined by the WADA test. A study by Moldakarimov et al. (2010) noted that reduction of induced gamma activity is associated with task-related priming in visual processing and is associated with improved perception. They suggested that this was to do with “representation sharpening” where neurons whose preferred features do not match the target stimulus were eliminated on repetition of the stimulus. The task used in this study involved a simple two-choice decision regarding the semantic or phonological characteristics of the target stimulus; it is possible that the arrival of the stimulus which resolved the choice was associated with the inhibition of the irrelevant option. Lachaux et al. (2008) using intracerebral EEGs have also reported gamma decreases associated with semantic and phonological processing of single words. Similarly the interpretation was in terms of deactivation of task-irrelevant areas.

Both the semantic and phonological tasks were associated with beta band power decreases, within the same time window, in the left middle temporal gyrus, in the region of BA37/BA39 (Figures 2A,B and 3A,B). With respect to semantic processing, this is in keeping with the suggestion that the left temporo-parietal-occipital junction (BA39), and in particular the angular gyrus, may play a role in semantic processing (Price, 2000; Patterson et al., 2007). The observed co-location with phonological processing in this area is not in accord with models based on lesion and spatial mapping studies, that locate such processing in the parietal areas (Paulesu et al., 1993). However, when techniques with finer temporal resolution are employed, activation of the temporal areas during phonological processing is reported (e.g., Indefrey and Levelt, 2004). Buchsbaum and D’Esposito (2008) suggest as well that phonological processing is also associated with activation of an area of “auditory-motor” interface in the temporal regions, although the temporal activation in our task is more posterior than that identified in their review. The closely similar spatio-temporal signatures of activation in this region for the two types of task would suggest that closely matched, if not identical, processes are occurring here. The slightly differing spectral characteristics of the activation may suggest otherwise though; the 20–30 Hz beta activity associated with the semantic task (Figure 2A) matches and precedes that shown in the parietal area (BA40) for this task (Figure 2C). Similarly, the 14–20 Hz beta activity associated with the phonological task matches and precedes in time that shown in the frontal area BA46. This could be interpreted as showing that these temporal areas are common to both phonological and semantic processing and the underlying networks, but that the *sequence* of activation in these networks and the frequencies involved differentiate the two types of task. Similar suggestions of this type of parallel processing are found in the work by Pammer et al. (2004), using MEG to track the time course of visual word recognition, where the middle temporal gyrus was co-active in parallel with the visual word form area, previously hypothesized to precede temporal activation.

In a similar fashion, in the parietal area BA40 we found beta band power decreases associated with both tasks (Figures 2C,D and 3C,D). However, power decreases in the phonological task (350–550 ms) preceded those in the semantic task (500–700 ms). Hemodynamic techniques would not have allowed discrimination at this level of temporal resolution, which may account for reports of both types of processing being associated with activation in this area, and which have led to models suggesting that semantic processing may also include accessing the phonological representation of words (Nobre and McCarthy, 1994; Matsumoto et al., 2005). The additional temporal and spectral information provided here suggests that the same network is involved but it is possible to segregate the two types of processing, while not disproving the possibility of functional overlap.

For frontal areas, both semantic and phonological task comparisons revealed a significant gamma power decrease within left superior/middle frontal regions (BA10 – Figures 2E,F and 3E,F). BA10 activation has been associated with access to semantic content (Demb et al., 1995; Binder et al., 1997). Blumenfeld et al. (2006) suggested that such frontal activation may be more associated with a (less efficient) feature selection process as opposed to (more efficient) access to an elaborated semantic representational system indexed by activation in the temporal areas. In the current semantic task, where participants showed 98.0% accuracy in the living/non-living task, the frontal activation occurred simultaneously with that in the temporal areas. There is less direct evidence of BA10 involvement with phonological processing, although Oh et al. (2011) report activation in the right BA10 area in a task manipulating phonological complexity. The gamma decreases reported by Lachaux et al. (2008) for a phonological task were also confined to the frontal areas and were interpreted as suppression of task-irrelevant areas. Previous reports of frontal activation in semantic processing have generally been associated with high-level semantic anomaly, sentence completion tasks, with reviews suggesting that frontal involvement occurred with greater task demands, such as context monitoring and complex response choices rather than semantic processing *per se* (Binder et al., 2009). As the task in this study was a relatively simple single word processing task, frontal activation was irrelevant to its satisfactory completion.

In this study we measured gamma in the 30–50 Hz range. Although focus on a range around 40 Hz is common in such research (e.g., Hannemann et al., 2007), other studies in this area have reported significant GBA associated with linguistic processing at higher frequencies, e.g., Kujala et al. (2011). Obleser and Kotz (2011), report GBA activity in both the 40 Hz range and higher (60–80 Hz), with both ranges showing significant variations as a factor of semantic context effects. In their study, the most robust findings were in the higher frequency band, although they generally paralled those in the 40 Hz range. Similarly, other studies examining GBA as indexing cognitive processing have reported higher frequency responses (e.g., 80–100 Hz) as indexing “attentional focusing” (Vidal et al., 2006) and “feature integration” (Morgan et al., 2011). It is possible that extending the range of GBA measured in studies attempting to deconstruct the contribution of overlapping cortical areas to different cognitive processes could allow even finer-grained analysis of the processing time-line.

In the IFG (BA45) a group level significant power reduction in the beta range (14–20 Hz) was found in the phonological task but not in the semantic task, although at the individual participant level there was evidence of power reductions in this area. The observed power decrease in the IFG (BA45) in the phonological task is consistent with fMRI findings (Paulesu et al., 1993; Poldrack et al., 1999; Costafreda et al., 2006); the failure to find this effect with the semantic task is not consistent with other studies reporting activation with semantic processing or models suggesting the involvement of both types of processing in this area (Hagoort, 2005). This may be due to a higher degree of spatial, temporal, and subject-demand variability associated with semantic processing (Maess et al., 2006). It has also been suggested that the semantic demands of living/non-living decision tasks may be insufficient to produce observable left inferior frontal effects (Roskies et al., 2001). Studies of gamma band responses to semantic tasks involving degraded speech report frontal and temporal involvement when the task involved sentence completion (Obleser and Kotz, 2011) but only temporal involvement when the task involved single word processing (Hannemann et al., 2007).

Overall, there was evidence of apparent co-location of both phonological and semantic processing in a network that includes left temporal, parietal, and frontal areas. Acknowledging the weaker spatial resolution of our approach, these processes were, however, discriminable using time-frequency characteristics that would not have been identified with hemodynamic techniques. The beta band changes in the IFG during phonological processing succeed those in the temporal areas, suggesting that the primed target resulted in early and automatic access to temporally based phonological representations (Matsumoto et al., 2005), with subsequent involvement of frontal areas for response preparation. BA10 activation is associated with gamma frequency in *both* tasks, which could be consistent with a suggestion that frontal areas are associated with the “unification” aspect of linguistic processing (Bastiaansen et al., 2010).

### Summary – spatio-temporal-spectral profiles of semantic and phonological processing

Both tasks elicited activity in frontal, temporal, and parietal areas, despite previous research assigning some form of dedication to phonological processing in the parietal areas and to semantic processing in the temporal regions, but in accord with models attributing a role, with the nature of that role sometimes disputed, to the frontal areas in both types of task (Hagoort, 2005; Grodzinsky and Santi, 2008; Willems and Hagoort, 2009). This is also coincident with previous fMRI findings. Consideration of the additional frequency and timing information obtained here allowed some separation/segregation of different aspects of the two types of task. It is possible that more contemporary approaches to fMRI analysis may allow a similar demonstration of functional specificity at the spatial level (Fedorenko et al., 2011) but this would still not offer the temporal segregation that can be offered by EEG/MEG techniques, nor the insights offered by examining oscillatory activity.

Comparison of patterns of beta activity can index the task-related relationship between access to “semantic” and to “phonological” stores, with gamma activity indexing the more generic “unification” aspect of linguistic processing demands (Bastiaansen et al., 2010). Additional timing information can track whether the different stages of the two tasks occur in parallel or in sequence, even when these involve the same cortical areas, as was the case for BA40 and BA10 in our study. The combined spatio-temporal and spectral information of MEG therefore allowed us to distinguish different roles for closely overlapping functional units and the sequence of their involvement in phonological and semantic processing.

Our aim in this paper was to demonstrate that, in instances where spatial imaging has been unable to segregate the neural correlates of processes that are clearly differentiated at the behavioral level, the addition of temporal measures, or combined temporal and frequency measures, could resolve such ambiguities. In the event, we feel that the combined MEG + SAM techniques reported here (a) illustrate the problem and (b) offer some resolution. Future studies could employ analyses using smaller and overlapping time windows and frequency bands which, although computationally challenging, will better demonstrate the evolution of these changes.

## Conflict of Interest Statement

The authors declare that the research was conducted in the absence of any commercial or financial relationships that could be construed as a potential conflict of interest.

## Footnotes

^1^www.ece.drexel.edu/ICVC/Align/align11.html

^2^http://www.jiscmail.ac.uk/lists/mri3dX.html
